# The Antiviral Effect of Indonesian Medicinal Plant Extracts Against Dengue Virus In Vitro and In Silico

**DOI:** 10.3390/pathogens8020085

**Published:** 2019-06-22

**Authors:** Rosmalena Rosmalena, Berna Elya, Beti E. Dewi, Fithriyah Fithriyah, Hidayati Desti, Marissa Angelina, Muhammad Hanafi, Puspa D. Lotulung, Vivitri D. Prasasty, Dimas Seto

**Affiliations:** 1 Department of Medical Chemistry, Faculty of Medicine, Universitas Indonesia, Depok, Jawa Barat 16424, Indonesia; rosmalena2018@gmail.com; 2 Faculty of Pharmacy, Universitas Indonesia, Depok, Jawa Barat 16424, Indonesia; berna.elya@farmasi.ui.ac.id; 3 Department of Microbiology, Faculty of Medicine, Universitas Indonesia-Cipto Mangukusumo Hospital, Jalan Pengangsaan Timur No. 16, Jakarta 10320, Indonesia; betied@yahoo.com (B.E.D.); titi_sjatha@yahoo.com (F.F.); hidayati.fkui@gmail.com (H.D.); 4 Research Centre for Chemistry LIPI, Kompleks Puspiptek, Serpong 15416, Indonesia; marissarfat@gmail.com (M.A.); hanafi124@yahoo.com (M.H.); puspade@yahoo.com (P.D.L.); 5 Faculty of Biotechnology, Atma Jaya Catholic University of Indonesia, Jalan Jenderal Sudirman 51, Jakarta 12930, Indonesia; vivitri.dewi@atmajaya.ac.id

**Keywords:** Plant extract, dengue virus, antiviral activity, *Acorus calamus*, *Cymbopogon citratus*, *Myristica fatua*

## Abstract

Dengue infections are still a worldwide burden, especially in Indonesia. There is no specific medication against the dengue virus. Recently, many types of research have been conducted to discover a new drug for dengue virus using natural resource extracts. Indonesia, as a tropical country, has a wide biodiversity. There are several medicinal plants in Indonesia that are believed to possess anti-dengue activity, such as *Myristica fatua*, *Cymbopogon citratus,* and *Acorus calamus* plants. We conducted an in vitro laboratory experiment of several extracts from Indonesian herbs combined with in silico analysis. The extracts were evaluated for safety and antiviral activity in Huh7it-1 cell lines, using a single dose of 20 µg/mL and dose-dependent (5, 10, 20, 40, 80 and 160 µg/mL) of plant extracts against dengue virus serotype 2 (DENV-2) NGC strain. The DMSO 0.1% was used as a negative control. The cytotoxic aspect was assessed by counting the cell viability, while the antiviral activity was calculated by counting the average inhibition. The selectivity index (SI) of plant extracts were performed from a ratio of CC_50_/EC_50_ value. In silico analysis was conducted to determine the free energy of binding between NS5 of dengue virus with bioactive compounds contained in *Myristica fatua*, *Cymbopogon citratus* and *Acorus calamus* extract plants. We determined that all extracts were not toxic against Huh7it-1 cell lines. The methanolic extracts of *A. calamus*, *C. citratus,* and *M. fatua* showed inhibition of DENV-2 at a dose of 20 µg/mL to 96.5%, 98.9%, and 122.7%, respectively. The dose-dependent effects showed that *M. fatua* has the best inhibition activity towards DENV-2. Molecular docking result showed that artesunic acid within *M. fatua* has the best free energy of binding (−7.2 kcal/mol), followed by homoegonol (−7.1 kcal/mol) which was slightly different from artesunic acid among others. The methanolic extracts of *A. calamus*, *C. citratus,* and *M. fatua* showed prospective anti-dengue activities both in vitro and in silico. Future research should be conducted to find the pure extracts of all useful herbs as a new candidate of antiviral drug.

## 1. Introduction

It has estimated that 390 million dengue infections occur worldwide annually [1]. Several outbreaks occurred almost in every place in the world, especially in the Asia region [2]. In Indonesia, there was approximately 800,000 total dengue cases and 200,000 hospitalized cases in 2015 [3]. Dengue virus consists of 4 serotypes, namely dengue virus serotype 1 (DENV-1), serotype 2 (DENV-2), serotype 3 (DENV-3), and serotype 4 (DENV-4), with all of them circulating in Indonesia [4,5]. Besides Indonesia, the other Asian countries where outbreaks of DENV have been substantial were Malaysia, the Philippines, Thailand, and Vietnam; while some Latin America locations including Brazil, Colombia, Honduras, Mexico, and Puerto Rico, were affected during 2011–2014. The burdens varied widely according to country, however, the rates were generally higher and the disease was more frequently severe in Asian countries than in Latin American countries [6]. 

Currently, there is no specific treatment for dengue infection. However, appropriate medical treatment could save the lives of patients [7]. Clinical manifestations of DENV infection range from asymptomatic: Dengue Fever (DF), Dengue Hemorrhagic Fever (DHF), ultimately Dengue Shock Syndrome (DSS) [8]. The preventive measures for mitigating dengue infection are mainly vector control and proposed dengue vaccine [6]. Research in dengue vaccine has shown some promise in preventing severe dengue cases but still has a long way to go [7]. One of the most critical issues of new antiviral drug development is the in vitro model experiment to be used. The cell substrate requirements for virus replication limit the screening of new compounds. For this reason, only a few drugs have arisen as efficient antiviral agents [9]. 

As an alternative, several medicinal plant extracts had been investigated to develop a new antiviral drug for dengue [8]. Some of the phytochemical compounds such as flavonoid, phenolics, and terpenoid that could be extracted from natural products were suggested to have an antiviral feature against dengue [10]. Indonesian plants such as *Curcuma longa* had been investigated as a potential antiviral against DENV and showed promising results [11]. The main objective of this study was to evaluate the effectiveness of plant extracts from *Myristica fatua*, *Acorus calamus*, and *Cymbopogon citratus* as antiviral agents against dengue virus infection in human Huh7it-1 cell lines in vitro and molecular docking in silico. The specific objectives were: i) to optimize the antiviral assay for dengue virus, ii) to measure the CC_50_ and EC_50_ of plant extracts in vitro, iii) to determine the selectivity index (SI) of plant extracts towards DENV, and iv) to predict the free energy of binding of antiviral agents with DENV protein target.

## 2. Results

The antiviral assay is essential to examine the maximum non-toxic concentration (MNTC) of the extract that is not toxic to the cells in the first step. After the MNTC of the extracts was assessed, 20 μg/mL of *A. calamus, C. citratus* and *M. fatua,* then were applied to Huh7it-1 cells infected by DENV. The result of Huh7it-1 cell lines treated with 20 µg/mL of three crude extracts showed different cytotoxic effects. Table 1 revealed that all of the extracts tested in this study were considered as being safe treatments with a range of cell viability in vitro assays that varied from 96.5% to 122.7%. Thus, it suggested that there was no significant cytotoxic effect on the Huh7 it-1 cell lines. 

The percentage of infectivity reduction of plant extracts and inhibition of DENV-2 NGC strain in Huh7it-1 cell was shown in Table 2. This result showed that the methanolic extract of *Myristica fatua* and *Cymbopogon citratus* gave over than 50% inhibition of DENV-2 NGC strain in vitro. Moreover, methanolic extract of three plant extracts with 20 µg/mL may vary in DENV infectivity and inhibition effects, with high viability of the cells (Table 1 and Table 2). Further research needs to be conducted to isolate and characterize the pure compounds from three those plant extracts with antiviral activities. 

To deepen investigation towards plant extract on antiviral aspects, DENV were treated with various concentration of extract before infected to the Huh7it-1 cells. The plant extract concentrations were used: 160 μg/mL, 80 μg/mL, 40 μg/mL, 20 μg/mL, 10 μg/mL, and 5 μg/mL, respectively. DMSO 0.1% was used as a negative control. To obtain assurance that plant extracts were not toxic to the cell, the half cytotoxic concentrations (CC_50_) were determined. This was achieved from the result of the MTT assay. The cell viability still showed a high level after being treated with plant extracts at concentrations of up to 40 µg/mL for *A. calamus* and *M. fatua* extracts, while *C. citratus* extract showed decreased cell viability after 20 µg/mL.

The other in vitro parameter has been defined in an attempt to quantify the effectiveness of antiretroviral agents, most importantly the 50% effective concentrations (EC_50_) as inhibition of viral replication or symptoms in an appropriate cell culture treatment of the disease. The viral replication inhibition increased as the three plant extract concentrations increased. This indicated that there were antiviral activities from those three plant extracts to DENV. From the ratio equation between CC_50_ and EC_50_, the SI of three plant extracts are shown on Table 3.

**Figure 1:**
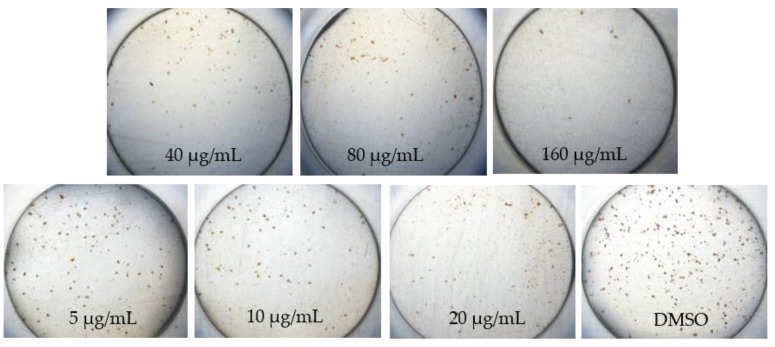
Morphological changes of DENV-1-infected Huh7it-1 cell lines treated with methanolic extracts of *Acorus calamus* at seven days post infection.

**Figure 2:**
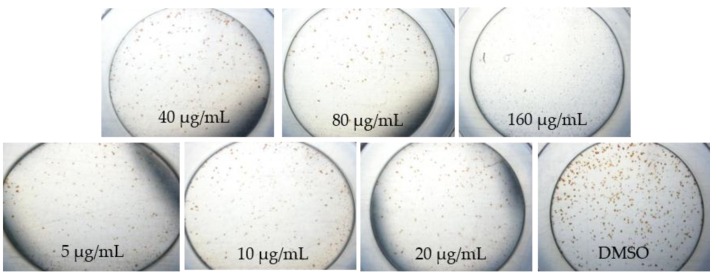
Morphological changes of DENV-1-infected Huh7it-1 cell lines treated with methanolic extracts of *Myristica fatua* at seven days post infection.

**Figure 3:**
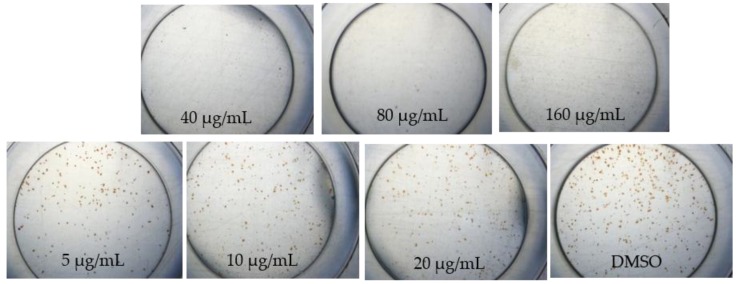
Morphological changes of DENV-1-infected Huh7it-1 cell lines treated with methanolic extracts of *Cymbopogon citratus* at seven days post infection.

Interestingly, the focus images of three plant extracts described different patterns on how to treat DENV in various concentrations. *A. calamus* (Figure 1) and *M. fatua* (Figure 2) extracts were able to maintain Huh7it-1 cell lines survival up to 80 µg/mL, while *C. citratus* (Figure 3) was not able to do so.

To explore more detail on the interaction between phytochemical constituents inside those three plant extracts and targeting DENV protein, molecular docking was conducted to simulate the potential inhibitors of three plant extracts against dengue virus by targeting the DENV protein that encoded protease enzyme DENV. The NS5 (Non-structural domain 5 of Methyltransferase) is an enzyme that roles multiple functions in the cytoplasm of infected cells, enabling viral RNA replication and counteracting host antiviral responses. Thus, it plays a critical role in interfering cellular splicing when DENV infects the host cells [12]. Through inhibiting this enzyme, some phytochemical constituents might be suggested as potential antiviral compounds. The molecular docking results are listed on Table 4, whereas nine constituents yielded various free energy of binding. It can be seen that artesunic acid was the best inhibitor based on its free energy of binding value (−7.2 kcal/mol) among others.

**Figure 4:**
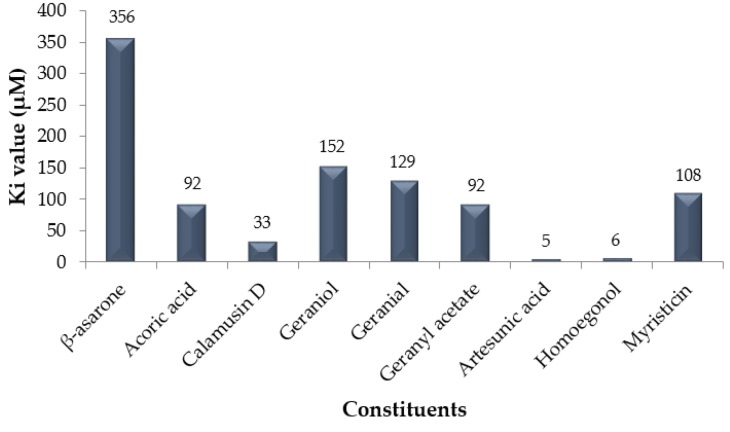
The inhibition constant (Ki) value in µM from nine constituents, where artesunic acid performed the best Ki value among others.

To find further inhibition activities of nine constituent, we also determined the constant inhibition value by converting the free energy of binding following the equation:

(1) ΔG = RT ln Ki,

From Figure 4 described that artesunic acid has the most efficient Ki value (5 µM) followed by homoegonol (6 µM) among others. The in silico results were equivalent with in vitro experiments, where *M. fatua* extract contains phytochemical constituents which have higher antiviral activities compared to phytochemical constituents in *A. calamus* and *C. citratus*.

**Figure 5:**
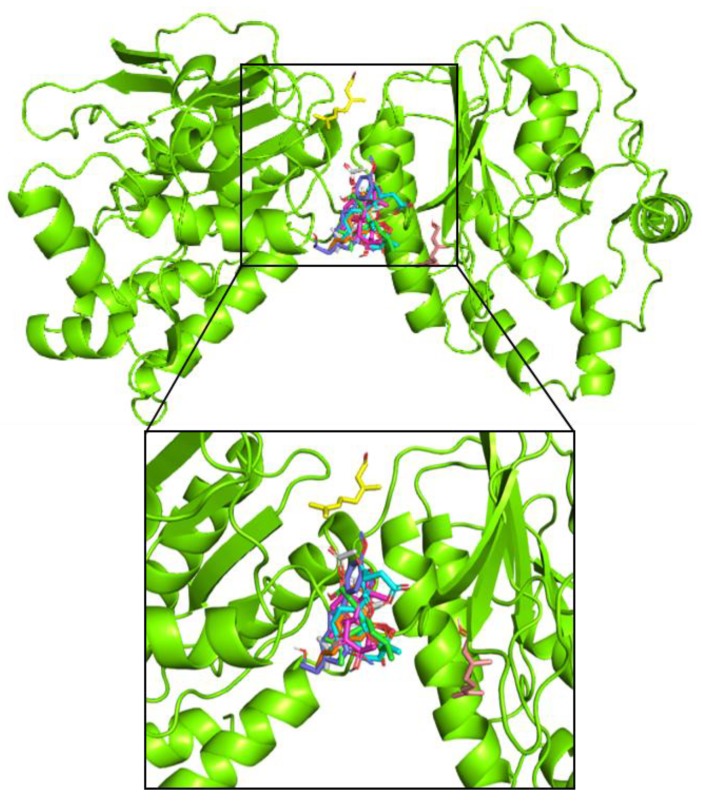
Binding interaction between NS5 protein of dengue virus with β-asarone (dark green), acoric acid (light green), calamusin D (magenta), geraniol (tints-wheat), geranial (yellow), geranyl acetate (white), artesunic acid (cyan), homoegonol (blue), myristicin (orange).

The structural binding of nine constituents towards NS5 protein DENV are depicted on Figure 5. It can be seen that binding interaction between NS5 protein of dengue virus is depicted using green with β-asarone in dark green, acoric acid in light green , calamusin D in magenta, geraniol in ints-wheat, geranial in yellow, geranyl acetate in white, artesunic acid in cyan, homoegonol in blue, and myristicin in orange. The binding mode eight constituents were at the same site with different poses, while the only geranial has a different binding site. 

## 3. Discussion

Exploration to find the antiviral drugs to DENV has been developing rapidly and profoundly in many countries. Herbal medicines are a potential source for the development of new antiviral drugs, since they can be selected based on their ethno-medicinal use, for example, against infectious diseases [13]. These plants produce a variety of phytochemical constituents with the potential to inhibit viral replication, and compounds from natural sources are of interested as possible sources to control viral infection [14]. The previous study showed that methanolic leaf extracts of *Hippophae rhamnoides*, sea-buckthorn from a family of Elaegnaceae is a potential candidate for dengue infection management [15]. Other herbal medicines such as *S. baicalensis* extract showed inhibition of DENV serotypes replication in vitro by reducing DENV infectivity and replication in Vero cells [16].

Our study revealed that a methanolic extract of *M. fatua* reduced the DENV infectivity to 21.61% with no cytotoxic effect on the viability of Huh7it-1 cell lines. Previous study showed that a component of the methanolic extract showed significantly the highest amount of flavonoid compound (332 mg quercetin equivalent [QE]/g), and the hexane extract showed the highest amount of phenolics by a significant amount (195 mg gallic acid equivalent [GAE]/mg) [17]. The other study using ethyl acetate extracts of testa and methanolic extracts of kernel and aril exhibited good antioxidant activities based on the IC_50_ values [17]. The minimum inhibitory concentration (MIC) assay revealed on the chloroform extract of aril at the concentration of 5 mg/mL was enough to inhibit the growth of bacteria *Staphylococcus aureus*. Flavonoids, such as quercetin and apigenin are the most studied flavonoids, which are known to be moderately correlated with antiviral activity as well as antibacterial and antioxidant activities [17,18].

Two neolignan compounds also well-recognized as resolcinols were isolated from the ethyl acetate fraction of the bark of *M. fatua* including malabaricone C and malabaricone B, which also possess known antiviral activities [19]. Other researchers reported novel cytotoxic lignan and diaryl nonanoid derivatives from the leaves of *M. fatua* [20,21]. Besides the action as antiviral agents, both compounds showed in vitro cytotoxic activity against the breast cancer cell lines MCF-7, with IC_50_ of 26.19 and 8.33 mM, respectively [18]. Recently, the same group also reported malabaricone B and C being successfully isolated from the stem bark of *M. fatua*. Lignans and acylphenols are the major secondary metabolite compounds of *M. fatua* leaf extract that produced by this family. In addition, their various health activities were also found to have anti-inflammatory, antioxidant, antibacterial, antimicrobial, nematocide, and cytotoxic effects [20]. 

Moreover, a previous study on the antiviral drug against DENV investigated 12 compounds of an ethanolic extract of *A. calamus* root, where tatanan A compound showed the best anti-DENV capacity and significantly alleviated DENV2-induced CPE and cytotoxicity effects, with an EC_50_ value of 3.9 µM. Time-addition assays showed that tatanan A affected the early stage of viral RNA replication, which in turn inhibited mRNA and protein levels of DENV2 [22].

Another crude extract in this study that inhibited DENV without any cytotoxic effect was *C. citratus* extract, with a reduction of infectivity to 47.8%. The compounds identified in *C. citratus* are mainly terpenes, alcohols, ketones, aldehyde, and esters. Some of the reported phytoconstituents are essential oils that contain citral α, citral β, nerol geraniol, citronellal, terpinolene, geranyl acetate, myrecene, and terpinol methylheptenone [23]. Essential oils and the most important compounds produced during the secondary metabolism in aromatic plants. Essential oils are volatile, have a characteristic odor, and are used as defensive agents by plants. In the previous study, all of the essential oils displayed notable inhibition against HSV-121. They also displayed antibacterial, antifungal, antioxidant, and insecticidal agents [24]. The compound of *C. citratus* is very encouraging and indicates that this herb should be studied more extensively to confirm antiviral activity to DENV.

The half cytotoxic concentration (CC_50_) value of *A. calamus*, *C. citratus* and *M. fatua* extracts were 424.93, 183.74, and 474.42 μg/mL, respectively. There was a viral inhibition, even though it showed dengue virus escalation at lower concentrations. This escalation might be useful for diagnosing patients, which can be utilized to increase and culture the virus of a dengue infected patient. The CC_50_ is a concentration of a substance that can be used to inhibit viral activity by 50%. Saptawati et al. reported that the other Indonesian plants: *Psidium guajava* and *Carica papaya* have shown high inhibitory activity towards dengue virus. The IC_50_ of them were 7.2 μg/mL and 6.57 μg/mL, respectively [25]. The obtained data in this study was analyzed by an unpaired t-test using a GraphPad Prism. It showed that the virus replication on the cells that were treated with three plant extracts at concentrations 160 μg/mL, 80 μg/mL, and 40μg/mL was significant (*p* value < 0.05) while the others did not show significant replication. The investigation of nine major active constituents as anti-dengue of three plant extracts are listed on Table 3. It was reported that these bioactive constituents have many pharmacological effects, such as antiviral properties [26,27,28,29]. 

Moreover, the obtained results of SI values presumed that the plant extracts are good for therapeutic use. The development of drugs including dengue antivirals from extracts of natural ingredients provides a better therapeutic effect compared to pure compound, so it is advantageous for the extraction process. This was shown in the study of the *Artemisia annua* plant as a anti-plasmodium for the treatment of malaria, where *A. annua* extract showed activity was 6 times better than the pure artemisinin compound contained in the plant [30]. This is related to the pharmacokinetic properties of the material, where *A. annua* is absorbed faster than artemisinin compounds. In addition, plant extract compounds are also capable of giving positive synergistic effects of all bioactive compound contained in the plant extract [31].

The morphological changes of DENV-1-infected Huh7it-1 cell lines treated with methanolic extracts of *M. fatua* at seven days post infection showed the most potency to inhibit DENV virus. This result is agreed with by infectivity and inhibition activities shown by methanolic extract of *M. fatua* in vitro, viability assay in vitro, and molecular docking in silico. Thus, we suggest that methanolic extract of *M. fatua* has the best antiviral activity against DENV.

Molecular docking was done by interacting non-structural domain 5 (NS5) protein of DENV with bioactive compounds which have known as antiviral activities, including β-asarone, acoric acid, and calamusin D which have found in *A. calamus*. Geraniol, geranial and geranyl acetate were found in *C. citratus*. At the same time, artesunic acid, homoegonol, and myristicin were found in *M. Fatua*. Docking results showed that artesunic acid had the highest free energy of binding (−7.2 kcal/mol) followed by homoegonol with slightly different free energy of binding (−7.1 kcal/mol) with artesunic acid among others.

## 4. Materials and Methods 

### 4.1. Extract Preparation

We prepared three extract samples from the above mentioned herbs plants, consist of methanolic extracts of *A. calamus*, *M. fatua*, and *C. citratus*. The extract preparation was conducted at the Department of Chemistry, Faculty of Medicine, Universitas Indonesia. Each extract was diluted in DMSO at a concentration of 100 mg/mL and stored at −80 °C until it was used.

### 4.2. Cell Culture and Virus

Propagation of DENV and antiviral assay in this study was done the same way as in the previous study with slight modification [16]. We used 96 well-plat flat bottom well plate for cytotoxicity and antiviral assays. In this study, we used DENV serotype two strain New Guinea C (NGC) and propagated in C6/36 adapted in human cell line of Huh-7 it-1 cell. A monolayer of Huh7it-1 cell in T-75 flasks were infected with DENV-2 NGC at a multiplicity of infection (MOI) of 0.5 FFU/cell and incubate it at 37 °C with 5% CO_2_ for 7 days as the previous study. The titer of DENV in the supernatant of C6/36 cell was checked by focus assay. The microscopic observation was done at 40x magnification after seven days post infection.

### 4.3. Determination of Cytotoxicity Assay

In vitro cytotoxicity effect was determined by viability of Huh7it-1 cell lines in 96 well-plate after treated with natural extract [16]. After 24 hours, the cells were treated with a single dose of 20 ug/mL and was further incubated at 37 °C, 5% CO_2_. After 48 hours, discard cell supernatant and 20 µL of 3-(4,5-Dimethylthiazol-2-yl)-2,5 diphenyl tetrazolium bromide (MTT) (Promega) salt solution was added into each well. Then, we incubated the cells for 4 hours according to the manufacturer’s instruction. The absorbance reading of each well was measured using a microplate reader at λ=490 nm. The percentage of cell viability were further determined based on the absorbance readings. The cell viability was determined as the percentage of cell viability to determine cytotoxic concentration (CC_50_). The CC_50_ value was obtained from nonlinear regression analysis of concentration-dependent curves by the graph and was represented as the means ± standard deviation experiments.

### 4.4. Determination of Infectivity Assay

In vitro infectivity assay was done in vitro. The 100 µl/well of Huh7it-1 was added at a density of 2 × 10 ^5^ cell/mL and incubated at 37 °C with 5% CO_2_ into 96 well-plate. After 24 hours, the cells were infected with DENV-2 at a MOI of 0.5 FFU/cell, which contained extracts of 20 µg/mL After 2 hours infection, we added 100 µL of DMEM +2% FBS that contained 20 µg/mL of the natural extract. Plates were further incubated at 37 °C for three days. Then we harvested to determine antiviral activity by counting the virus titer with focus assay with a slight modification [16]. Briefly, the mixtures of 10-fold serial dilution of viral supernatant were inoculated onto Huh7it-1 cell monolayer in triplicate wells. Adsorption was carried out at 35 °C in 5% CO_2_ for 2 hours with agitation at 30 minute intervals. Methylcellulose 1.5% overlay medium was added, and infected cells were incubated at 35 °C in 5% CO_2_ for two days. Staining of infected cells was done according to the previous study. The result from Focus assay was used to determine the effective concentration (EC_50_) based on infectivity percentage. The EC_50_ was obtained from nonlinear regression analysis of concentration-dependent curves by the graph and represented as the means ± standard deviation experiments.

### 4.5. The Selectivity Index Analysis 

The 50% cytotoxic (CC_50_) and 50% inhibition (IC_50_) concentrations were calculated from concentration-dependent curves. The results were obtained from triplicate experiments with at six extract concentrations. The percentage of cytotoxicity was calculated as [(A − B)/A] × 100, where A and B are the OD 540 nm of untreated and of treated cells, respectively. The percentages of inhibition were calculated as [(A − B) × 100/(C − B)], where A, B and C indicate the absorbance in 540 nm of the extracts, virus and cell controls, respectively. Each obtained EC_50_ value was defined as the effective concentration that reduced the absorbance of infected cells to 50% when compared with cell and virus controls. The CC_50_ and IC_50_ of each compound were obtained from dose-dependent curves (not shown). The CC_50_ and IC_50_ were the average of independent triplicate experiments with six concentrations within the inhibitory range of the compounds. The selective index (SI) was defined as CC_50_/EC_50_ [32].

### 4.6. Molecular Docking Analysis

The molecular docking preparation was done by using the 3D crystal structure of DENV NS5 (PDB ID: 4R8R) retrieved from Protein Database (https://www.rcsb.org/). The removal of all crystallographic water molecules of NS5 structure was done. The final NS5 structure was saved in the pdbqt format for further molecular docking analysis. The preparation 3D structures of antiviral compound datasets were retrieved from PubChem (https://pubchem.ncbi.nlm.nih.gov/) in sdf format and converted into pdb formats by translation webserver (https://cactus.nci.nih.gov/translate/). The nine molecules of datasets were then saved in pdbqt files to further molecular docking analysis using Autodock Vina [33,34]. 

## 5. Conclusions

Methanolic extract of *A. calamus*, *C. citratus,* and *M. fatua* have antiviral effects to DENV without any cytotoxic effect. Further studies are needed to investigate the IC_50_, CC_50_ value, and those extracts mechanism of antiviral action. In addition, purification and characterization of compounds are needed to define the active compounds of antiviral DENV. The CC_50_ value of *A. calamus*, *C. citratus,* and *M. fatua* extracts were 424.93 μg/mL, 183.74 μg/mL and 474.42 μg/mL. The EC_50_ value of *A. calamus*, *C. citratus,* and *M. fatua* extracts were not detected, 29.37 μg/mL and 25.33 μg/mL. The SI values of *A. calamus*, *C. citratus,* and *M. fatua* extracts were not detected. The molecular docking analyses showed that artesunic acid and homoegonol were the two best compounds in inhibiting NS5 protein DENV based on their free energy of binding and Ki values.

## Figures and Tables

**Table 1:** Viability of selected plant extracts on Huh7it-1 cells at a single dose of 20 µg/mL.

Methanolic Extract	Cell Viability (%)
*Acorus calamus*	96.5
*Cymbopogon citratus*	98.9
*Myristica fatua*	122.7

**Table 2:** Percentage of infectivity and inhibition of DENV-2 on Huh7it-1 cell lines.

Sample	Average Infectivity (%)	Average Inhibition (%)
*Acorus calamus* (leaves methanol extract)	73.4	26.6
*Cymbopogon citratus* (root methanol extract)	47.8	52.2
*Myristica fatua* (methanol extract)	21.6	78.4

**Table 3:** The 50% cytotoxic (CC_50_) and 50% inhibition (IC_50_) concentrations, and selective index (SI) of plant extracts against DENV on Huh7it-1 cell lines.

Plant Extract	CC_50_ (µg.mL^−1^)	EC_50_ (µg.mL^−1^)	SI
*Acorus calamus*	424.93	n.d.*	n.d.
*Cymbopogon citratus*	183.74	29.37	6.26
*Myristica fatua*	474.42	25.33	18.73

n.d. * = not detected

**Table 4:** Molecular docking results between antiviral agents and NS5 of dengue virus.

No.	Constituent	Plant Source	Free energy of Binding (kcal/mol)	References
1	β-asarone	*Acorus calamus*	−4.7	26
2	Acoric acid	*Acorus calamus*	−5.5	26
3	Calamusin D	*Acorus calamus*	−6.1	26
4	Geraniol	*Cymbopogon citratus*	−5.2	27
5	Geranial	*Cymbopogon citratus*	−5.3	28
6	Geranyl acetate	*Cymbopogon citratus*	−5.5	28
7	Artesunic acid	*Myristica fatua*	−7.2	29
8	Homoegonol	*Myristica fatua*	−7.1	29
9	Myristicin	*Myristica fatua*	−5.4	29
